# Estresse, Mulheres e Infarto Agudo do Miocárdio: O que se Sabe?

**DOI:** 10.36660/abc.20200968

**Published:** 2020-10-13

**Affiliations:** 1 Hospital Universitário Clementino Fraga Filho Rio de JaneiroRJ Brasil Hospital Universitário Clementino Fraga Filho, Rio de Janeiro, RJ – Brasil; 2 Universidade Federal do Rio de Janeiro Brasil Universidade Federal do Rio de Janeiro – UFRJ – Brasil

**Keywords:** Doenças Cardiovasculares, Mulheres, Infarto do Miocárdio, Estresse Psicológico, Doença Arterial Coronária

Durante décadas as mulheres foram excluídas da pesquisa em saúde. Este fato foi objeto de crítica do *National Institutes of Health* (NIH),^1^ que buscou encorajar a inclusão de mulheres em ensaios clínicos de pesquisa, com parco progresso nos anos 1980. Alguns dos exemplos mais conhecidos aconteceram na pesquisa cardiovascular. O *Harvard Physicians Health Study,*^2^ que analisou a relação entre o uso moderado de aspirina e doenças cardíacas, não incluiu mulheres na amostra estudada. Na ocasião, o resultado do estudo não pôde ser conclusivo para a sua aplicação em mulheres. The *Multiple Risk Factor Intervention Trials* (MR. FIT)^3^ foi outra grande pesquisa sobre doença cardíaca que também não incluiu indivíduos do sexo feminino. Este estudo nacional examinou como os níveis de colesterol, pressão arterial e tabagismo afetavam o desenvolvimento de doença cardíaca.

A omissão das mulheres destas e de outras pesquisas à época merece destaque, tendo em vista as taxas de mortalidade cardiovascular entre as mulheres.^4^

A história da civilização ocidental mostra a predominante dominação masculina dentro e fora do lar, como a mulher era submissa, e os papéis que a ela eram reservados, quais sejam, responsabilidades da casa e da família.^5^ A busca de espaço profissional na sociedade moderna tornou a mulher protagonista no mercado de trabalho, embora a maioria delas não tenha conseguido deixar de exercer a segunda jornada no retorno à casa.

A *World Health Organization* (WHO)^6^ apresenta no *Atlas of Heart Disease and Stroke*, no tópico *Cardiovascular Disease*, entre outros assuntos, os fatores de risco e assinala entre os *Other modifiable risk factors* o estresse psicossocial (“estresse crônico da vida, isolamento social e ansiedade”), que aumenta o risco de doença cardíaca e acidente vascular cerebral. Igualmente, refere “depressão” como associada àquele aumento. Neste atlas, o ítem 12 é intitulado *Women: a special case*? Ali assinala *Risks for women only*: “uso de contraceptivos orais; terapia de reposição hormonal; síndrome dos ovários policísticos; maior risco de ataque cardíaco no início de cada ciclo menstrual”. A mulher carrega consigo tais singularidades e também as tensões experimentadas na administração da vida pessoal, familiar e profissional que lhe geram sobrecarga emocional, apesar de ter havido avanços na atenção e cuidados para a mulher nesse cenário das doenças cardiovasculares. Bom que assim seja, porque naquele capítulo está escrito que as mulheres se equivocam ao achar que são mais propensas ao câncer do que às doenças cardiovasculares.

O artigo “Um olhar sobre o estresse nas mulheres com Infarto Agudo do Miocárdio”^7^ estudou uma amostra que também incluiu homens e usou um instrumento com quatro estágios (alerta, resistência, quase-exaustão e exaustão). A fase de “alerta”, com as reações do sistema nervoso autônomo ao estressor; “resistência”, fase em que o indivíduo busca modos de lidar com o estresse; e as fases de “quase-exaustão” e “exaustão”, que são caracterizadas pelo início do processo de adoecimento tendo como alvo os órgãos mais vulneráveis ou quando, efetivamente, as doenças se manifestam. Nesse estudo^7^ ficou evidente a maior susceptibilidade das mulheres ao sofrimento pelo estresse, pois pontuaram o dobro dos homens nas fases de “quase-exaustão” (32,9% x 16,7%) e de “exaustão” (18,6% x 9,2%).

O estresse psicossocial deve ser estudado usando-se uma medida composta, pois ocorrem várias manifestações na esfera subjetiva, biológica e comportamental, justificando-se uma abordagem integrada. Um estudo^8^ que avaliou sintomas de depressão, ansiedade, raiva, estresse geral percebido, estresse pós-traumático e hostilidade e usou instrumentos específicos, estudou uma amostra de mulheres com doença coronariana estável, prospectivamente. Os autores padronizaram as escalas e combinaram em um índex composto para analisarem estatisticamente. Encontraram mulheres com alto nível de estresse psicológico e que tiveram significantemente maior incidência de eventos cardiovasculares. A medida de níveis de estresse percebido também é muito usada em pacientes com infarto agudo do miocárdio (IAM) e um estudo observou que mulheres relataram mais altos níveis de estresse do que os homens no período de 12 meses pós-IAM.^9^ Não obstante, outro importante estudo prospectivo (18 anos de seguimento) investigou em uma grande amostra de mulheres e homens a associação entre a percepção do impacto do estresse sobre a saúde autoavaliada e a incidência de doença arterial coronariana (DAC).^10^ Os investigadores observaram que os participantes que responderam “muito ou extremamente” tiveram aumentado o risco de DAC. O novo aspecto está na avaliação do impacto do estresse. Esse estudo predisse a incidência de DAC independente da escala de níveis de estresse percebido e os autores afirmam que é razoável assumir que a única questão “Até que ponto você acha que o estresse ou a pressão em sua vida afetou sua saúde?” pode ser usada em *settings* de cuidado geral ou especializado.

Por fim, ainda há muito a saber sobre stress psicológico e doença cardiovascular. É uma pauta que tem muito a ser estudada e, a considerar a pergunta do estudo acima, sugerimos pesquisas que usem método qualitativo e possam ouvir as vozes das pessoas, independente de sexo, em contraponto e como complemento às inúmeras investigações com método de pesquisa quantitativo.

## References

[1] 1. U.S. General Accounting Office, National Institutes of Health: Problems in Implementing Policy on Women in Study Populations. Statement of Mark V. Nadel, Associate Director of National and Public Health Issues, Human Resources Division, before the Subcommittee on Health and the Environment, Committee on Energy and Commerce, U.S. House of Representatives (GAO/T-HRD-90-80), 1990

[2] 2. Steering Committee of the Physicians Health Study Research Group: Final report on the aspirin compornent of the on-going physicians health study. N Engl J Med.1989; 321:129-35. 10.1056/NEJM198907203210301 2664509

[3] 3. Blumenthal SJ, Barry P, Hamilton J. Forging a Women's Health Research Agenda. Washington, DC: National Women's Health Resource Center; 1991.

[4] 4. Johnson TL, Fee E. Women's Health Research: An Introduction, in Women's Health Research: In: Haseltine FP, Jacobson BG, (editors). A Medical Policy Primer. Washington, DC: Publisher Unknown; 1997.

[5] 5. Costa FA. Mulher, Trabalho e Família: Os impactos do trabalho na subjetividade da mulher e em suas relações familiares. Pretextos. 2018; 3(6):434-52.

[6] 6. Mackay J, Mensah GA, Mendis S, Greenlund K. The Atlas of Heart Disease and Stroke [internet]. Brighton (UK): World Health Organization, (WHO); 2004 [cited 2020 Aug 23]. Part One, Cardiovascular Disease; p. 16-21. Available from: https://www.who.int/cardiovascular_diseases/resources/atlas/en/

[7] 7. Schmidt K, Lima AS, Schmitt KR, Moraes MA, Schmidt MM. Stress in Women with Acute Myocardial Infarction: A Closer Look. Arq Bras Cardiol. 2020; 115(4):649-657. 10.36660/abc.20190282 PMC8386975 33111864

[8] 8. Pimple P, Lima BB, Hammadah M, Wilmot K, Ramadan R, Levantsevych O,et al. Psychological Distress and Subsequent Cardiovascular Events in Individuals With Coronary Artery Disease. J Am Heart Assoc. 2019;8(9):9. 10.1161/JAHA.118.011866 PMC6512132 31055991

[9] 9. Xu X, Bao H, Strait KM, Edmondson DE, Davidson KW, Beltrame JF, et al. Perceived stress after Acute Myocardial Infarction: A Comparison between Young and Middle-Aged Women Versus Men. Psychosom Med. 2017;79(1):50-8. 10.1097/PSY.0000000000000429 PMC5459399 27984507

[10] 10. Nabi H, Kivimäki M, Batty GD, Shipley MJ, Britton A, Brunner EJ, et al. Increased risk of coronary heart disease among individuals reporting adverse impacto of stress on their health: The Whitehall II prospective cohort study. Eur Heart J.34(4):2697-705. 10.1093/eurheartj/eht216 PMC3766148 23804585

