# Supplementary material for: Estresse, Mulheres e Infarto Agudo do Miocárdio: O que se Sabe?
Source: Arq Bras Cardiol. 2020 Oct 13;115(4):658–9. [Article in Portuguese] doi: 10.36660/abc.20200968 (PMC8386979; doi:10.36660/abc.20200968)
Supplement: Translation

# Translation: Stress, Women and Acute Myocardial Infarction: What is known?

**Affiliations:** 1 Rio de JaneiroRJ Brazil Hospital Universitário Clementino Fraga Filho, Rio de Janeiro, RJ – Brazil; 2 Brazil Universidade Federal do Rio de Janeiro – UFRJ – Brazil

**Keywords:** Cardiovascular Diseases, Women, Myocardial Infarction, Psychological Stress

For decades, women were excluded from health research. This fact has been criticized by the National Institutes of Health (NIH),^1^ which sought to encourage the inclusion of women in clinical research trials, with little progress in the 1980s. Some of the most well-known examples occurred in cardiovascular research. The Harvard Physicians Health Study,^2^ which analyzed the relationship between moderate use of aspirin and heart disease, did not include women in the sample studied. In that occasion, the result could not be conclusive for its application to women. The Multiple Risk Factor Intervention Trials (MR. FIT)^3^ was another major research on heart disease that did not include female individuals. This national study examined how cholesterol levels, blood pressure and smoking affected the development of heart disease.

The omission of women from these and other studies at the time deserves to be highlighted in view of the cardiovascular mortality rates among women.^4^

The history of Western civilization shows the predominant male domination inside and outside home, how women were submissives, and the roles that were reserved for them, namely, domestic and family responsibilities.^5^ The search for a professional space in modern society has made women protagonists in the job market, although most of them have not been able to stop playing the second round when they are back home after a busy workday.

The World Health Organization (WHO)^6^ presents in the Atlas of Heart Disease and Stroke, in the topic Cardiovascular Disease, among other subjects, the risk factors and points out among the *Other modifiable risk factors* psychosocial stress (“chronic life stress, social isolation and anxiety”) which increases the risk of heart disease and stroke. Likewise, it mentions “depression” as associated with that increase. In this atlas, item 12 is entitled *Women: a special case?* There, *Risks for women only* quotes: “use of oral contraceptives; hormone replacement therapy; polycystic ovary syndrome; greater risk of heart attack at the beginning of each menstrual cycle”. Women carry with them such singularities and also the tensions experienced in the management of personal, familiar and professional life that generate emotional overload, despite advances in attention and care for women in this scenario of cardiovascular diseases. Good to be so, because in that chapter it is written that women are wrong to think that they are more prone to cancer than to cardiovascular diseases.

The article “Stress in women with Acute Myocardial Infarction: A close look”^7^ studied a sample that also included men and used an instrument with four stages (alertness, resistance, near-exhaustion and exhaustion). The “alert” phase, with the reactions of the autonomic nervous system to the stressor; “resistance”, the individual looks for ways to deal with stress; and the “near-exhaustion” and “exhaustion” phases, that are characterized by the beginning of the process of illness targeting the most vulnerable organs or when, effectively, the diseases manifest themselves. In this study,^7^ the greater susceptibility of women to suffering from stress was evident, as they scored twice as many men in the “near-exhaustion” (32.9% x 16.7%) and “exhaustion” (18.6% x 9.2%).

Psychosocial stress should be studied using a composite measure, as there are several manifestations in the subjective, biological and behavioral sphere, justifying an integrated approach. A study^8^ that evaluated symptoms of depression, anxiety, anger, perceived general stress, post-traumatic stress and hostility and used specific instruments, prospectively studied a sample of women with stable coronary disease. The authors standardized the scales and combined them into a composite index to analyze them statistically. They found women with a high level of psychological stress and who had a significantly higher incidence of cardiovascular events. The measure of perceived stress levels is also widely used in patients with acute myocardial infarction (AMI) and a study found that women reported higher levels of stress than men in the 12-month period after an AMI.^9^ Nevertheless, another important prospective study (18 years of follow-up) investigated in a large sample of women and men the association between the perception of the impact of stress on self-rated health and the incidence of coronary artery disease (CAD).^10^ This study predicted CAD incidence regardless of the perceived stress level scale and the authors claim that it is reasonable to assume that the only question “To what extend do you feel that the stress or pressure you have experienced in your life has affected your health?” can be used in general or specialized care settings.

Finally, there is still a lot to know about psychological stress and cardiovascular disease. It is a subject that has a lot to be studied and, considering the question of the study above, we suggest researches to use qualitative method and to listen to people's voices, regardless of sex, in counterpoint and as a complement to the countless investigations with quantitative research method.
